# Enhancement of Acetate-Induced Apoptosis of Colorectal Cancer Cells by Cathepsin D Inhibition Depends on Oligomycin A-Sensitive Respiration

**DOI:** 10.3390/biom14040473

**Published:** 2024-04-12

**Authors:** Sara Alves, Cátia Santos-Pereira, Cláudia S. F. Oliveira, Ana Preto, Susana R. Chaves, Manuela Côrte-Real

**Affiliations:** CBMA—Centre of Molecular and Environmental Biology, Department of Biology, University of Minho, 4710-057 Braga, Portugal; sara.csa@gmail.com (S.A.); catia91pereira@gmail.com (C.S.-P.); csfoliveira@ucp.pt (C.S.F.O.); apreto@bio.uminho.pt (A.P.)

**Keywords:** colorectal cancer, short-chain fatty acids, acetate, apoptosis, cathepsin D, cell respiration

## Abstract

Colorectal cancer (CRC) is a leading cause of death worldwide. Conventional therapies are available with varying effectiveness. Acetate, a short-chain fatty acid produced by human intestinal bacteria, triggers mitochondria-mediated apoptosis preferentially in CRC but not in normal colonocytes, which has spurred an interest in its use for CRC prevention/therapy. We previously uncovered that acetate-induced mitochondrial-mediated apoptosis in CRC cells is significantly enhanced by the inhibition of the lysosomal protease cathepsin D (CatD), which indicates both mitochondria and the lysosome are involved in the regulation of acetate-induced apoptosis. Herein, we sought to determine whether mitochondrial function affects CatD apoptotic function. We found that enhancement of acetate-induced apoptosis by CatD inhibition depends on oligomycin A-sensitive respiration. Mechanistically, the potentiating effect is associated with an increase in cellular and mitochondrial superoxide anion accumulation and mitochondrial mass. Our results provide novel clues into the regulation of CatD function and the effect of tumor heterogeneity in the outcome of combined treatment using acetate and CatD inhibitors.

## 1. Introduction

Colorectal cancer (CRC) is a growing public health concern, and prevention and early detection remain the best options to reduce mortality [1]. Indeed, CRC is a significant health burden and one of the most prevalent cancers worldwide. Despite the advances in CRC treatment options, classical chemotherapy with 5-Fluorouracil (5-FU) is still a clinical problem due to resistance, presenting response rates of less than 10% in metastatic CRC patients [2]. The intestinal microbiota, composed of a large population of microorganisms modulated by dietary patterns, has been increasingly linked with CRC [3,4]. Differences in intestinal microbiota composition have been reported between patients with CRC and healthy individuals, and the reduced production of microbiota-derived short-chain fatty acids (SCFAs), namely acetate, butyrate, and propionate, is linked to a high CRC risk [5,6]. Therefore, several authors have proposed using propionibacteria as probiotics in CRC treatment/prevention [7,8,9]. However, the effectiveness of this approach would likely vary according to individual intestinal flora and dietary compliance. In the search for new alternatives for CRC treatment capable of overcoming some of the limitations associated with the currently available approaches in the clinic, SCFA administration has been also been considered [3,7,8,10,11,12]. This possibility was advanced due to their described role in several biological processes against CRC cells [13,14,15]. However, its widespread use requires detailed elucidation of the molecular pathways involved.

In the past, Jan and colleagues specifically focused on the antitumoral activity of acetate, having already demonstrated that this metabolite can inhibit proliferation and induce a well-characterized mitochondria-mediated pathway preferentially in CRC cells [8]. We also showed that acetate interferes with energetic metabolism through the modulation of monocarboxylate transporter (MCT) expression [15]. More recently, we demonstrated that a mixture of SCFAs in physiological proportions can modulate several biological processes in CRC cells, namely cell survival, proliferation, apoptosis, energetic metabolism, cytosolic pH, and lysosomal membrane permeabilization (LMP) [3]. These results highlight the possibility of applying SCFAs in the clinic as potentiators of classical chemotherapy, thus promoting the quality of life of patients by decreasing its associated side effects.

Mechanistically, we found that acetate promotes LMP and the consequent release of cathepsin D (CatD), but not of cathepsins B or L. We described a novel anti-apoptotic role for this lysosomal protease [13,14], which is often overexpressed in CRC [16,17], since we found that acetate-induced apoptosis of CRC cells was enhanced by the inhibition of CatD, either with siRNA or pepstatin A (PstA), which was associated with higher mitochondrial dysfunction and increased mitochondrial mass [14]. We further described that autophagy is inhibited during acetate-induced apoptosis of CRC cells, suggesting that cytosolic CatD provides the conditions for degradation of damaged mitochondria, directly or indirectly, through a process alternative to autophagy but with a similar protective role. We thus proposed that CatD inhibitors could enhance acetate-mediated CRC cell death. Notably, the data obtained with CRC cell lines mimicked our previous results with the yeast *Saccharomyces cerevisiae* Pep4p, a vacuolar protease ortholog to human CatD, which we showed translocates from the vacuole to the cytosol following vacuolar membrane permeabilization and has a pro-survival role during mitochondria-dependent apoptosis induced by acetic acid [13,18]. We also showed that autophagy is not active in cells undergoing acetic acid-induced apoptosis, and that Pep4p has a role in mitochondrial degradation during this process that depends on proteolytic activity [13,18]. However, we later uncovered that Pep4p can also play opposing roles in acetic acid-induced apoptosis depending on the yeast cellular background: a protective role in *S. cerevisiae* W303-1A and an executioner role in *S. cerevisiae* BY4741 [18,19]. Since the BY4741 strain has a reduced respiratory capacity and a lower mitochondrial mass than the W303-1A strain [20,21], we hypothesized that mitochondrial respiratory activity might affect the protective role of Pep4p in acetic acid-induced cell death. Indeed, the deletion of *PEP4* in W303 respiratory-deficient (rho0) cells resulted in a higher resistance to acetic acid-induced cell death, in contrast to what was observed in *rho*^+^ cells [19]. To exclude the pleiotropic effects of mitochondrial DNA depletion that do not account for respiratory deficiency, we used oligomycin A. This Streptomyces-specific macrolide disrupts the coupling between the F_1_ and F_0_ subunits of the F_1_F_0_-ATPase complex, blocking proton conductance across the synthase complex and inhibiting the synthesis of mitochondrial ATP by oxidative phosphorylation [22]. The hindrance of proton translocation by the components of the electron transport chain, due to the high proton gradient generated, ultimately results in decreased respiration. Altogether, these results suggested that the pro-survival role of Pep4p in acetic acid-induced apoptosis is dependent on oligomycin A-sensitive respiration. In fact, when mitochondrial respiration is inhibited, Pep4p can have a role in the execution of yeast cell death rather than in cell protection.

Although most cancer-related pre-clinical studies have assessed how gene expression and/or malignant mutations affect resistance to therapy [23,24,25], other efforts have addressed how the altered metabolism of tumor cells can aid in the fight against cancer [26]. Indeed, many cancer cells, including CRC cells, display a metabolic shift towards aerobic glycolysis over oxidative phosphorylation, which has been named the Warburg effect [27]. However, it has become increasingly apparent that tumors are metabolically heterogeneous with cells displaying complex metabolic systems. The regulation of tumor metabolism can be affected by several factors, such as oxygen availability and glucose concentration, as well as other conditions in the tumor microenvironment like acidity and metabolite availability [28]. While the glycolytic metabolism of cancer cells has often been linked to resistance to treatment, it also provides opportunities in the fight against cancer. Indeed, many modern diagnostic techniques take advantage of imaging metabolic alterations in cancer cells [29]. Moreover, specific features of cancer cells which distinguish them from normal cells present good targets that can be exploited in therapeutic approaches, either in standalone or combination therapy [30].

In this study, we sought to assess whether oligomycin A-sensitive respiration affects the role of human CatD in acetate-induced apoptosis of CRC cells. We show that similarly to yeast cells, the enhancement of acetate-induced apoptosis of CRC cells by CatD inhibition depends on oligomycin A-sensitive respiration. While the results obtained provide new insights into the role of CatD in acetate-induced apoptosis of CRC cells, which may be exploited for the design of novel therapeutic strategies for CRC, they also reveal limitations in its application in the context of tumor heterogeneity.

## 2. Materials and Methods

### 2.1. Cell Lines and Culture Conditions

RKO cells (IPATIMUP, Porto, Portugal) were grown in DMEM (Biowest, Nuaillé, France) with a high-glucose solution supplemented with 1 mM of sodium pyruvate and 1.5 g/L of sodium bicarbonate and maintained at 37 °C under a humidified atmosphere containing 5% of CO_2_. Cells were seeded and adhered onto sterile plates 24 h before treatments. Cells were exposed to etoposide (50 µM) or H_2_O_2_ (1 mM) as controls, or a half-maximal inhibitory concentration (IC_50_) of acetate (110 mM) for 48 h [14]. When used, cells were pre-incubated with 100 µM of Pepstatin A (PstA) for 16 h and then co-incubated with the same concentration of PstA, with or without 110 mM of acetate, for 48 h. When specified, cells were also incubated with 1.25 µg/mL of Oligomycin A for 48 h. Etoposide, H_2_O_2_, PstA, and Oligomycin A were purchased from Merck KGaA, Darmstadt, Germany.

### 2.2. Sulforhodamine (SRB) Assay

The SRB assay was performed as previously described [14]. Briefly, cells were fixed in ice-cold methanol containing 1% (*v*/*v*) of acetic acid and then incubated for 1.5 h at 37 °C with 0.5% (*w*/*v*) of SRB dissolved in 1% (*v*/*v*) of acetic acid. Then, cells were washed with 1% (*v*/*v*) of acetic acid and SRB was solubilized with 10 mM of Tris, with a pH of 10. The absorbance was read at 540 nm in a microplate reader (SpectraMax 340PC, Molecular Devices, Sunnyvale, CA, USA). All samples were measured in triplicate and the results were normalized in relation to the negative control (cells incubated only with fresh completed medium), considered as 100% of cell proliferation.

### 2.3. Determination of O_2_ Consumption

After 48 h, cells were trypsinized, centrifuged (1300× *g*, 5 min), and resuspended in 1 mL of fresh medium. The rate of O_2_ consumption was then assessed for each condition using a Clark electrode previously calibrated with fresh medium. The rate in the absence of oligomycin A was considered as 100%.

### 2.4. Caspase-3 Activity

Cells were collected and processed as described previously [14]. After treatments, floating and attached cells were collected, then washed twice with 1x PBS, and lysed in a Lysis Buffer (10 mM Tris, pH 7.5, 0.1 M NaCl, 1 mM EDTA, 0.01% (*v*/*v*) Triton X-100) through multiple freeze/thaw cycles. A total of 50 μg of the total extracts (1 mg/mL) were then diluted in a 1:2 ratio with 200 μM of z-DEVD-AFC (Biomol, Plymouth Meeting, PA, USA) in a 2 × reaction buffer (20 mM PIPES, pH 7.4, 4 mM EDTA, 10 mM DTT). The fluorescence of the cleaved 7-amino-4-trifluoromethyl coumarin (AFC) from DEVD-AFC (Biomol, Plymouth Meeting, PA, USA) was measured using a microplate reader (Fluoroskan Ascent FL, Thermo Scientific Inc., Waltham, MA, USA).

### 2.5. Flow Cytometry

After treatments, approximately 1 × 10^6^ floating and attached cells were collected, washed with 1× PBS, centrifuged at 1500× *g* for 5 min, and incubated with the appropriate probes for 30 min in the dark, as follows: 150 nM of dihydroethidium (DHE, Molecular Probes, Eugene, OR, USA) (37 °C) to detect the superoxide anion (O_2_.^−^), 2.5 μM of MitoSOX^TM^ Red (Molecular Probes, Eugene, OR, USA) (RT) for mitochondrial superoxide detection, or 400 nM of MitoTracker^®^ Green FM (Molecular Probes, Eugene, OR, USA) (37 °C) to analyze mitochondrial mass. Fluorescence emission was analyzed by flow cytometry using the following fluorescence channels: oxidized DHE using FL-4, oxidized MitoSOX^TM^ Red using FL-3, and MitoTracker^®^ Green using FL-1. For O_2_.^−^ measurements, values were expressed as the percentage of cells with positive staining normalized to T0 (the control for mitochondrial and total O_2_.^−^ levels before the treatment). For the mitochondrial mass analysis, values were expressed as the mean green fluorescence intensity normalized to that at T0. Flow cytometry assays were performed in an Epics^®^ XL™ (BeckmanCoulter, Brea, CA, USA) flow cytometer equipped with an argon-ion laser emitting a 488 nm beam at 15 mW. Thirty thousand cells per sample were analyzed. Data were analyzed with Flowing software (version 2.5.1, Turku Centre for Biotechnology, Turku, Finland).

### 2.6. Statistical Analysis

Data are expressed as the mean ± S.D. of three independent experiments. Statistical analysis was determined by a one-way ANOVA followed by a Dunnett or Bonferroni’s test for multiple comparisons with GraphPad Prism 5.0 software (GraphPad Software, Solana Beach, CA, USA). *p*-values < 0.05 were considered statistically significant.

## 3. Results

### 3.1. The Protective Role of Cathepsin D in Acetate-Induced Apoptosis Depends on Oligomycin A-Sensitive Respiration

**Figure 1:**
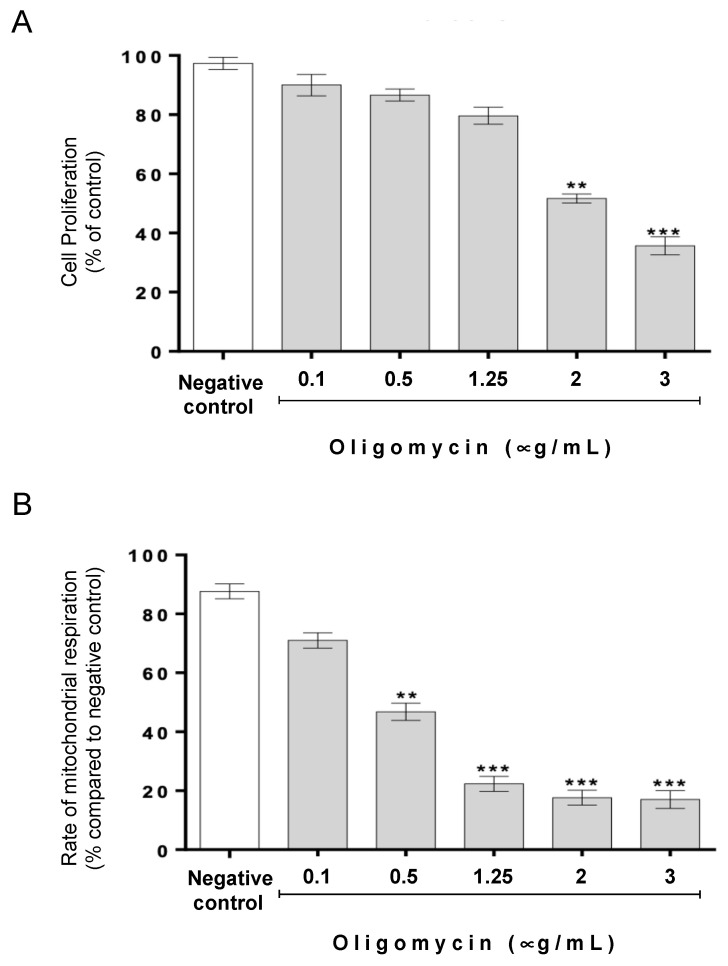
Effect of oligomycin A on cell proliferation and mitochondrial respiration of CRC cells. RKO cells were exposed to the indicated different concentrations of oligomycin A for 48 h. As a negative control, cells were grown only with fresh medium. (**A**) Cell proliferation was assessed by Sulforhodamine B (SRB assay). (**B**) The rate of mitochondrial respiration was assessed by estimating O_2_ consumption. (**A**,**B**) Values are expressed as the mean ± S.D. of at least three independent experiments. ** *p* < 0.1 and *** *p* < 0.001 compared with the negative control.

We have previously demonstrated that CatD protects CRC cell lines from apoptosis induced by acetate concentrations in the range of those found in the human intestinal tract [14]. Here, we assessed whether oligomycin A-sensitive respiration affects the role of CatD in this cell death process. As oligomycin A is cytotoxic to cell lines with varying sensitivities [31], we first optimized a non-cytotoxic concentration range. We found that up to 1.25 μg/mL oligomycin A had no significant effect on cell proliferation, as assessed by the SRB assay (Figure 1A). However, 1.25 μg/mL of oligomycin A caused an 80% decrease in oxygen consumption, indicating a significant reduction in the rate of mitochondrial respiration (Figure 1B).

**Figure 2:**
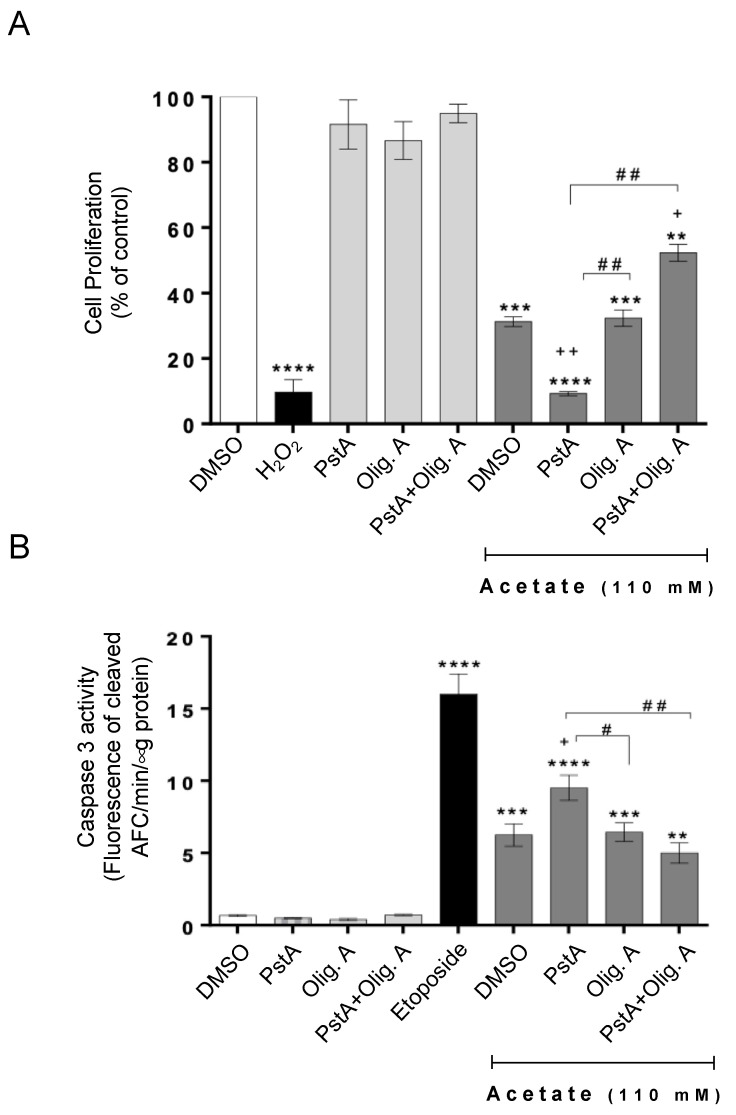
The protective role of CatD against acetate-induced apoptosis in CRC cells depends on oligomycin A-sensitive respiration. (**A**,**B**) RKO cells were exposed to acetate (0 or 110 mM) for 48 h plus DMSO, PstA, oligomycin A, or PstA plus oligomycin A. (**A**) Cell proliferation was determined by the SRB assay. H_2_O_2_ (1 mM) was the positive control. (**B**) Caspase-3 activity was determined by measuring the cleavage of DEVD-AFC in whole-cell extracts. Etoposide (50 µM) was the positive control. (**A**,**B**) ** *p* < 0.1, *** *p* < 0.001, and **** *p* < 0.0001 compared to the DMSO control. ^+^ *p* < 0.5 and ^++^ *p* < 0.1 compared to acetate/DMSO. ^#^ *p* < 0.5 and ^##^ *p* < 0.1 compared to acetate/PstA.

Next, we determined if inhibiting respiration with oligomycin A affected the acetate-induced inhibition of proliferation and/or apoptosis induction. We found that the exposure of RKO cells to acetate resulted in decreased proliferation and increased caspase activation, as previously reported, and that the presence of oligomycin A had no significant additional effect (Figure 2). To address whether the presence of CatD affects this phenotype, we inhibited its activity with PstA, previously shown to be equivalent to the downregulation of CatD by siRNA [13,14]. We found that PstA significantly reduced the proliferation of RKO cells exposed to acetate in the absence of oligomycin A, as we previously reported. In contrast, PstA increased the proliferation of cells exposed to acetate in the presence of oligomycin A (Figure 2A). Accordingly, while PstA greatly increased acetate-induced caspase-3 activation, the same was not observed in the presence of oligomycin A (Figure 2B). These results indicate that the effect of CatD can be modulated by oligomycin A-sensitive respiration. Indeed, we found that decreasing respiratory activity abrogates the previously described enhancement of acetate-induced apoptosis of CRC cells imparted by CatD inhibition.

### 3.2. The Role of Cathepsin D in Mitochondrial Degradation Is Not Affected by Oligomycin A-Sensitive Respiration

**Figure 3:**
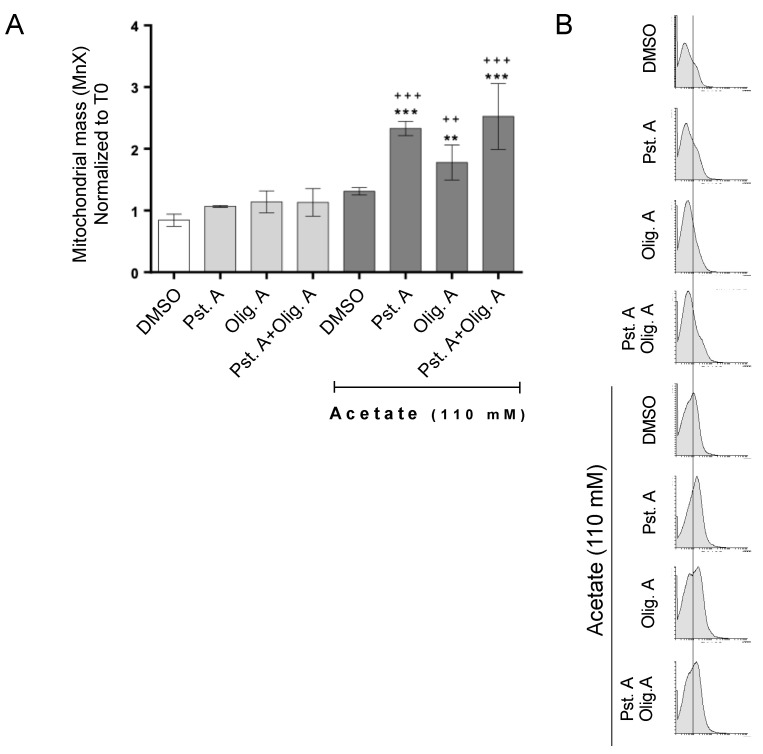
The role of CatD in mitochondrial degradation in CRC cells undergoing acetate-induced apoptosis is not affected by their respiratory status. RKO cells were exposed to acetate (0 or 110 mM) for 48 h plus DMSO, PstA, oligomycin A, or PstA plus oligomycin. Mitochondrial mass was assessed by flow cytometry of cells stained with MitoTracker^®^ Green FM. (**A**) Values represent the mean ± S.D of green mean fluorescence intensity (FL-1) normalized to T0. ** *p* < 0.1 and *** *p* < 0.001 compared to the DMSO control. ^++^ *p* < 0.1 and ^+++^ *p* < 0.001 compared to acetate/DMSO. (**B**) Representative histograms of green fluorescence intensity (counts in Y axis vs. FL1 log in X axis) corresponding to RKO cells treated as described in (**A**).

We previously showed that CatD is necessary for the efficient autophagic-independent degradation of mitochondria, which could underlie its protective role in acetate-induced apoptosis [13]. However, in the presence of oligomycin, CatD did not protect cells from either inhibition of proliferation or enhanced caspase-3 activity triggered by acetate, indicating that the role of this protease in mitochondrial degradation could be affected when oligomycin A-sensitive respiration is hindered. We therefore assessed mitochondrial mass by staining cells with Mitotracker^®^ Green FM, which has been used as a structural mitochondrial probe [32]. The inhibition of CatD with PstA resulted in a similar increase in mitochondrial mass, whether cells were exposed to acetate in the presence or absence of oligomycin A (Figure 3). Taken together, these data show that CatD is required for the efficient degradation of mitochondria in cells exposed to acetate regardless of their respiratory status.

### 3.3. Oligomycin A Decreases ROS Accumulation Induced by Acetate Independently of Cathepsin D Activity

**Figure 4:**
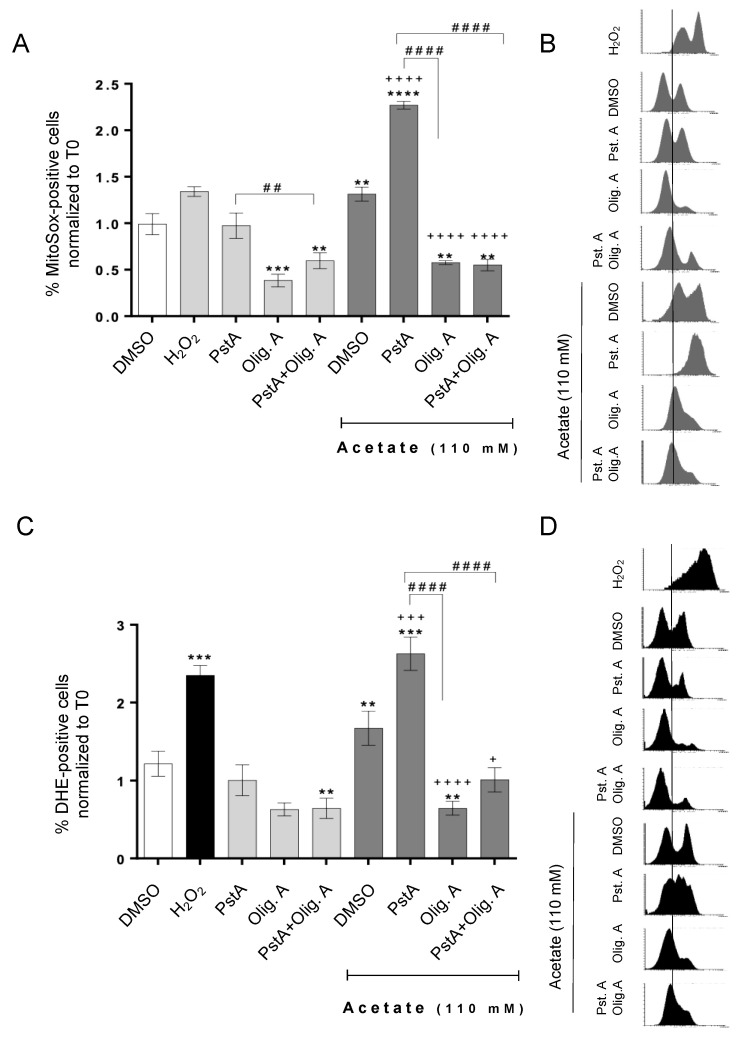
Oligomycin A decreases acetate-induced superoxide anion accumulation in CRC cells, regardless of CatD activity. RKO cells were exposed to acetate (0 or 110 mM) for 48 h plus DMSO, PstA, oligomycin A, or PstA plus oligomycin A. H_2_O_2_ (1 mM) was used as a positive control for ROS production. (**A**) The accumulation of the mitochondrial superoxide anion (O_2_.^−^) was assessed by flow cytometry of cells stained with MitoSOX Red. Values represent the mean ± S.D of the percentage of MitoSOX Red-stained cells (FL-3) after 48 h normalized to T0. (**B**) Representative histograms of red fluorescence intensity (counts in Y axis vs. FL3 log in X axis) corresponding to RKO cells treated as described in (**A**). (**C**) The accumulation of the superoxide anion (O_2_.^−^) was assessed by flow cytometry of cells stained with dihydroethidium (DHE). Values represent the mean ± S.D of the percentage of DHE-stained cells (FL-4) after 48 h normalized to T0. (**D**) Representative histograms of red fluorescence intensity (counts in Y axis vs. FL3 log in X axis) corresponding to RKO cells treated as described in (**C**). (**A**,**C**) ** *p* < 0.1, *** *p* < 0.001, and **** *p* < 0.0001 compared to the DMSO control. ^+^ *p* < 0.5, ^+++^ *p* < 0.001 and ^++++^ *p* < 0.0001 compared to acetate/DMSO. ^##^ *p* < 0.1 and ^####^ *p* < 0.0001 compared to acetate/PstA.

In the previous sections, we show that even though oligomycin A prevented the increase in acetate-induced apoptosis resulting from CatD inhibition, it did not reduce the accumulation of mitochondria in those cells. We therefore postulated that mitochondrial degradation would not be required to protect cells from acetate-induced apoptosis in oligomycin A-treated cells because mitochondrial damage could be lower. To confirm this hypothesis, we assessed the levels of both total and mitochondrial superoxide anion in cells under different conditions, tested by staining cells with DHE or MitoSOX^TM^ Red, respectively. Indeed, oligomycin A significantly reduced the accumulation of total and mitochondrial superoxide in response to acetate (Figure 4). Moreover, while PstA greatly increased both total and mitochondrial superoxide anion accumulation in cells exposed to acetate in the absence of oligomycin A, it had no effect in cells exposed to acetate in the presence of oligomycin A. These results suggest that, indeed, ROS accumulation is reduced in cells treated with oligomycin A, and thus degradation of mitochondria mediated by CatD is likely unnecessary to protect these cells from apoptosis induced by acetate. This reinforces our previous hypothesis that CatD protects cells from acetate-induced apoptosis by degrading damaged mitochondria. This role is apparent in respiring cells; however, a pro-apoptotic role of CatD is observed in poorly respiring cells exposed to acetate.

## 4. Discussion

CatD is an important apoptosis regulator, both under physiological and pathological conditions [33,34]. Depending on the cell type and context, CatD can induce or inhibit apoptosis, acting through different mechanisms [35,36,37,38,39]. Multiple studies have therefore addressed how to target this protease in cancer treatment, supported by the fact that CatD is often found overexpressed in several tumors [40,41,42,43,44,45]. In the past, we used two model systems, yeast and CRC-derived cell lines, to unravel the function of CatD in regulated cell death in response to acetic acid/acetate, which triggers a similar mitochondria-dependent apoptotic pathway with the involvement of the vacuole/lysosome [13,18,46]. Indeed, it was in yeast that we first uncovered that Pep4p, the yeast CatD, is released from the vacuole to the cytosol following vacuolar membrane permeabilization. This release of Pep4p protects cells from mitochondria-dependent apoptosis induced by acetic acid in a manner depending on its proteolytic activity [18,19]. We also showed that Pep4p plays an autophagy-independent role in mitochondrial degradation during this process, which also depends on its proteolytic activity [13,18,46]. Moreover, heterologously expressed human CatD complemented the function of Pep4p in acetic acid-induced apoptosis, indicating they share a conserved function [13]. As the deletion of *PEP4* in a different yeast background (BY4741) confers resistance to acetic acid, in contrast with the sensitivity previously observed in the W303 background, we hypothesized that this was due to altered mitochondrial mass between the W303 and BY strains, the latter of which has an insertion of a transposon in the *HAP1* gene that gives it an altered mitochondrial phenotype [47]. We then tested this hypothesis, and found that the deletion of *PEP4* in respiratory-deficient W303 (rho0) cells increased resistance to acetic acid-induced cell death [19]. Accordingly, in the presence of oligomycin A, W303 *pep4*Δ cells became more resistant to acetic acid-induced cell death than wild-type cells [19].

Considering that yeast behaves as an alternative cell model to CRC cells in the context of cell death induced by acetic acid/acetate, we aimed, as a proof of concept, to validate in RKO cells the dependence of the enhancement of acetate-induced apoptosis of colorectal cancer cells by Cat D inhibition on oligomycin A-sensitive respiration, as observed for acetic acid-induced apoptosis in yeast cells. We showed that the enhancement of acetate-induced apoptosis imparted by CatD inhibition depends on oligomycin A-sensitive respiration since PstA no longer increased acetate-induced proliferation inhibition or apoptosis induction in the presence of oligomycin A. However, mitochondrial mass still increased under the same conditions, indicating that an accumulation of mitochondria is no longer detrimental to cells exposed to acetate in poor-respiring cells. One possibility was that exposure to oligomycin A decreased mitochondrial damage, bypassing the need for CatD in mitochondrial removal. Indeed, we found that oligomycin A did not affect CatD-mediated mitochondrial degradation but decreased the accumulation of mitochondrial reactive oxygen species independently of CatD activity, indicating that this hypothesis was correct. Taken together, our data on RKO and yeast cells indicate that the pro-survival role of CatD/Pep4p in acetate/acetic acid-induced apoptosis is conserved and depends on oligomycin A-sensitive respiration, and that it is possible to tune the function of this protease pharmacologically. In fact, when mitochondrial respiration is decreased/inhibited, CatD/Pep4p may have a role in the execution of cell death rather than in cell protection. Notably, the proteolytic activity of Pep4p in the BY4741 background, where it has a pro-apoptotic role in acetic acid-induced apoptosis, is also required for mitochondrial degradation, as observed for W303-1A cells [19].

Cellular metabolism has taken center stage in cancer research, as it is well established that cancer cells mainly rely on glycolysis and carry out lactic acid fermentation even when oxygen is available [27,48]. However, even within the same tumor, different responses can be observed [48]. In the context of tumor metabolic heterogeneity, the addition of CatD inhibitors to acetate-based CRC combined treatment would enhance the elimination of cells more dependent on respiration but may have the opposite effect on glycolysis-dependent cells. For this reason, combination strategies with acetate and CatD inhibitors would likely benefit from the inclusion of metabolic modulators to increase respiration and/or ROS production. Several studies have investigated reversing metabolic reprogramming to specifically target cancer cells. For instance, the overexpression of human frataxin resulted in the induction of oxidative metabolism in the human colon carcinoma cell lines MIP101, DLD2, and HT29, but not in non-tumor cells, which was associated with a decrease in cell growth and tumor-forming ability, although without increased ROS production [49]. Another study reported that the inhibition of pyruvate dehydrogenase kinase 1 (PDK1) with dichloroacetate (DCA) can restore pyruvate dehydrogenase activity, as well as oxidative phosphorylation, in several cancer-derived cell lines, such as A549 (non-small-cell lung cancer), M059K (glioblastoma), and MCF-7 (breast cancer), associated with increased ROS production and apoptosis in A549 cells [50]. On the other hand, it is also worth noting that increased aerobic glycolysis, while beneficial for rapid growth, is not ideal to sustain the slow proliferating state characteristic of cancer stem cells/chemo-resistant cancer cells. In fact, several reports indicate that a metabolic shift towards an increased respiratory metabolism is characteristic of cancer stem cells and is also related to an acquired resistance to therapy [51]. For instance, it has been reported that CRC cells resistant to chronic 5-fluorouracil (5-FU) treatment shift their metabolism towards oxidative phosphorylation [52]. These data, and our data, suggest that a CRC therapy regimen using acetate plus CatD inhibitors alone or in combination with metabolic modulators may be effectively explored in the elimination of more- or less-respiratory-dependent cancer cells, respectively. Since the results obtained in our previous studies with yeast and acetic acid-induced cell death were validated by us and others in different CRC cell lines, namely RKO and HCT-15 cells (reviewed in [42]), we expect that the dependence of the enhancement of acetate-induced apoptosis by CatD inhibition on oligomycin A-sensitive respiration we observed is not specific to RKO cells. Nonetheless, additional studies with different CRC cells will be required to ensure the relevance of combination CRC therapy strategies with acetate, CatD inhibitors (both natural and synthetic [34]), and metabolic regulators, which should be explored in future research.

In summary, we uncovered that the role of CatD in apoptosis depends on cellular metabolic status. Our results therefore suggest that using CatD inhibitors simultaneously with acetate in CRC treatment may have opposite effects on individual cells undergoing acetate-induced apoptosis according to their dependence on mitochondrial metabolism. This provides novel clues into the function of CatD and supports the notion that personalized therapy should take into account both the genomic makeup and the metabolic status of tumor cells.

## Data Availability

Dataset available on request from the authors.

## Abbreviations

CRC	colorectal cancer;
CatD	cathepsin D;
DHE	dihydroethidium;
OligA	oligomycin A;
PstA	pepstatin A;
ROS	reactive oxygen species;
RT	room temperature
SCFA	short-chain fatty acids;
SRB	sulforhodamine B

## References

[1] Morgan E., Arnold M., Gini A., Lorenzoni V., Cabasag C.J., Laversanne M., Vignat J., Ferlay J., Murphy N., Bray F. (2023). Global burden of colorectal cancer in 2020 and 2040: Incidence and mortality estimates from GLOBOCAN. Gut.

[2] Blondy S., David V., Verdier M., Mathonnet M., Perraud A., Christou N. (2020). 5-Fluorouracil resistance mechanisms in colorectal cancer: From classical pathways to promising processes. Cancer Sci..

[3] Gomes S., Baltazar F., Silva E., Preto A. (2022). Microbiota-Derived Short-Chain Fatty Acids: New Road in Colorectal Cancer Therapy. Pharmaceutics.

[4] Kim J., Lee H.K. (2022). Potential Role of the Gut Microbiome In Colorectal Cancer Progression. Front. Immunol..

[5] Alvandi E., Wong W.K.M., Joglekar M.V., Spring K.J., Hardikar A.A. (2022). Short-chain fatty acid concentrations in the incidence and risk-stratification of colorectal cancer: A systematic review and meta-analysis. BMC Med..

[6] Peluzio M.D.C.G., Martinez J.A., Milagro F.I. (2021). Postbiotics: Metabolites and mechanisms involved in microbiota-host interactions. Trends Food Sci. Technol..

[7] Fotiadis C.I., Stoidis C.N., Spyropoulos B.G., Zografos E.D. (2008). Role of probiotics, prebiotics and synbiotics in chemoprevention for colorectal cancer. World J. Gastroenterol..

[8] Jan G., Belzacq A.-S., Haouzi D., Rouault A., Métivier D., Kroemer G., Brenner C. (2002). Propionibacteria induce apoptosis of colorectal carcinoma cells via short-chain fatty acids acting on mitochondria. Cell Death Differ..

[9] Lan A., Bruneau A., Bensaada M., Philippe C., Bellaud P., Rabot S., Jan G. (2008). Increased induction of apoptosis by Propionibacterium freudenreichii TL133 in colonic mucosal crypts of human microbiota-associated rats treated with 1,2-dimethylhydrazine. Br. J. Nutr..

[10] Comalada M., Bailón E., de Haro O., Lara-Villoslada F., Xaus J., Zarzuelo A., Gálvez J. (2006). The effects of short-chain fatty acids on colon epithelial proliferation and survival depend on the cellular phenotype. J. Cancer Res. Clin. Oncol..

[11] Scheppach W., Bartram H., Richter F. (1995). Role of short-chain fatty acids in the prevention of colorectal cancer. Eur. J. Cancer.

[12] Mowat C., Dhatt J., Bhatti I., Hamie A., Baker K. (2023). Short chain fatty acids prime colorectal cancer cells to activate antitumor immunity. Front. Immunol..

[13] Oliveira C.S.F., Pereira H., Alves S., Castro L., Baltazar F., Chaves S.R., Preto A., Côrte-Real M. (2015). Cathepsin D protects colorectal cancer cells from acetate-induced apoptosis through autophagyindependent degradation of damaged mitochondria. Cell Death Dis..

[14] Marques C., Oliveira C.S.F., Alves S., Chaves S.R., Coutinho O.P., Côrte-Real M., Preto A. (2013). Acetate-induced apoptosis in colorectal carcinoma cells involves lysosomal membrane permeabilization and cathepsin D release. Cell Death Dis..

[15] Ferro S., Azevedo-Silva J., Casal M., Côrte-Real M., Baltazar F., Preto A. (2016). Characterization of acetate transport in colorectal cancer cells and potential therapeutic implications. Oncotarget.

[16] Shin I.Y., Sung N.Y., Lee Y.S., Kwon T.S., Si Y., Lee Y.S., Oh S.T., Lee I.K. (2014). The expression of multiple proteins as prognostic factors in colorectal cancer: Cathepsin D, p53, COX-2, epidermal growth factor receptor, C-erbB-2, and Ki-67. Gut Liver.

[17] Sebzda T., Saleh Y., Gburek J., Andrzejak R., Gnus J., Siewinski M., Grzebieniak Z. (2005). Cathepsin D expression in human colorectal cancer: Relationship with tumour type and tissue differentiation grade. J. Exp. Ther. Oncol..

[18] Pereira C., Chaves S., Alves S., Salin B., Camougrand N., Manon S., Sousa M.J., Côrte-Real M. (2010). Mitochondrial degradation in acetic acid-induced yeast apoptosis: The role of Pep4 and the ADP/ATP carrier. Mol. Microbiol..

[19] Pereira H.P. (2015). The Role of Pep4p, the Vacuolar Yeast Protease Ortholog of Human Cathepsin D, in Mitochondria-Dependent Apoptosis. Ph.D. Thesis.

[20] Ocampo A., Liu J., Schroeder E.A., Shadel G.S., Barrientos A. (2012). Mitochondrial respiratory thresholds regulate yeast chronological life span and its extension by caloric restriction. Cell Metab..

[21] Devin A., Dejean L., Beauvoit B., Chevtzoff C., Avéret N., Bunoust O., Rigoulet M. (2006). Growth yield homeostasis in respiring yeast is due to a strict mitochondrial content adjustment. J. Biol. Chem..

[22] Antoniel M., Giorgio V., Fogolari F., Glick G., Bernardi P., Lippe G. (2014). The Oligomycin-Sensitivity Conferring Protein of Mitochondrial ATP Synthase: Emerging New Roles in Mitochondrial Pathophysiology. Int. J. Mol. Sci..

[23] Aghagolzadeh P., Radpour R. (2016). New trends in molecular and cellular biomarker discovery for colorectal cancer. World J. Gastroenterol..

[24] Palma S., Zwenger A.O., Croce M.V., Abba M.C., Lacunza E. (2016). From molecular biology to clinical trials: Toward personalized colorectal cancer therapy. Clin. Color. Cancer.

[25] Lipsyc M., Yaeger R. (2015). Impact of somatic mutations on patterns of metastasis in colorectal cancer. J. Gastrointest. Oncol..

[26] Vyas S., Zaganjor E., Haigis M.C. (2016). Mitochondria and Cancer. Cell.

[27] Lu J., Tan M., Cai Q. (2015). The Warburg effect in tumor progression: Mitochondrial oxidative metabolism as an anti-metastasis mechanism. Cancer Lett..

[28] Elia I., Haigis M.C. (2021). Metabolites and the tumour microenvironment: From cellular mechanisms to systemic metabolism. Nat. Metab..

[29] Sengupta D., Pratx G. (2016). Imaging metabolic heterogeneity in cancer. Mol. Cancer.

[30] Zhao Y., Butler E.B., Tan M. (2013). Targeting cellular metabolism to improve cancer therapeutics. Cell Death Dis..

[31] Salomon A.R., Voehringer D.W., Herzenberg L.A., Khosla C. (2000). Understanding and exploiting the mechanistic basis for selectivity of polyketide inhibitors of F_0_F_1_-ATPase. Proc. Natl. Acad. Sci. USA.

[32] Cottet-Rousselle C., Ronot X., Leverve X., Mayol J. (2011). Cytometric assessment of mitochondria using fluorescent probes. Cytom. Part A.

[33] Yadati T., Houben T., Bitorina A., Shiri-Sverdlov R. (2020). The Ins and Outs of Cathepsins: Physiological Function and Role in Disease Management. Cells.

[34] Mijanovic O., Petushkova A.I., Brankovic A., Turk B., Solovieva A.B., Nikitkina A.I., Bolevich S., Timashev P.S., Parodi A., Zamyatnin A.A. (2021). Cathepsin D—Managing the Delicate Balance. Pharmaceutics.

[35] Johansson A.C., Steen H., Öllinger K., Roberg K. (2003). Cathepsin D mediates cytochrome c release and caspase activation in human fibroblast apoptosis induced by staurosporine. Cell Death Differ..

[36] Bidère N., Lorenzo H.K., Carmona S., Laforge M., Harper F., Dumont C., Senik A. (2003). Cathepsin D triggers Bax activation, resulting in selective apoptosis-inducing factor (AIF) relocation in T lymphocytes entering the early commitment phase to apoptosis. J. Biol. Chem..

[37] Minarowska A., Minarowski Ł., Karwowska A., Gacko M. (2007). Regulatory role of cathepsin D in apoptosis. Folia Histochem. Cytobiol..

[38] Sagulenko V., Muth D., Sagulenko E., Paffhausen T., Schwab M., Westermann F. (2008). Cathepsin D protects human neuroblastoma cells from doxorubicin-induced cell death. Carcinogenesis.

[39] Di Y.-Q., Han X.-L., Kang X.-L., Wang D., Chen C.-H., Wang J.-X., Zhao X.-F. (2021). Autophagy triggers CTSD (cathepsin D) maturation and localization inside cells to promote apoptosis. Autophagy.

[40] Masson O., Bach A.-S., Derocq D., Prébois C., Laurent-Matha V., Pattingre S., Liaudet-Coopman E. (2010). Pathophysiological functions of cathepsin D: Targeting its catalytic activity versus its protein binding activity?. Biochimie.

[41] Gyrd-Hansen M., Nylandsted J., Jäättelä M. (2004). Heat shock protein 70 promotes cancer cell viability by safeguarding lysosomal integrity. Cell Cycle.

[42] Leto G., Tumminello F.M., Crescimanno M., Flandina C., Gebbia N. (2004). Cathepsin D expression levels in nongynecological solid tumors: Clinical and therapeutic implications. Clin. Exp. Metastasis.

[43] Benes P., Vetvicka V., Fusek M. (2008). Cathepsin D-Many functions of one aspartic protease. Crit. Rev. Oncol./Hematol..

[44] Palermo C., Joyce J.A. (2008). Cysteine cathepsin proteases as pharmacological targets in cancer. Trends Pharmacol. Sci..

[45] Basu S., Cheriyamundath S., Gavert N., Brabletz T., Haase G., Ben-Ze’ev A. (2019). Increased expression of cathepsin D is required for L1-mediated colon cancer progression. Oncotarget.

[46] Pereira H., Oliveira C.S.F., Castro L., Preto A., Chaves S.R., Côrte-Real M. (2015). Yeast as a tool to explore cathepsin D function. Microb. Cell.

[47] Gaisne M., Bécam A.M., Verdière J., Herbert C.J. (1999). A ‘natural’ mutation in Saccharomyces cerevisiae strains derived from S288c affects the complex regulatory gene HAP1 (CYP1). Curr. Genet..

[48] Chaube B., Malvi P., Singh S.V., Mohammad N., Meena A.S., Bhat M.K. (2015). Targeting metabolic flexibility by simultaneously inhibiting respiratory complex I and lactate generation retards melanoma progression. Oncotarget.

[49] Schulz T.J., Thierbach R., Voigt A., Drewes G., Mietzner B., Steinberg P., Pfeiffer A.F.H., Ristow M. (2006). Induction of oxidative metabolism by mitochondrial frataxin inhibits cancer growth: Otto Warburg revisited. J. Biol. Chem..

[50] Bonnet S., Archer S.L., Allalunis-Turner J., Haromy A., Beaulieu C., Thompson R., Lee C.T., Lopaschuk G.D., Puttagunta L., Bonnet S. (2007). A Mitochondria-K+ Channel Axis Is Suppressed in Cancer and Its Normalization Promotes Apoptosis and Inhibits Cancer Growth. Cancer Cell.

[51] Wolf D.A. (2014). Is Reliance on Mitochondrial Respiration a ‘Chink in the Armor’ of Therapy-Resistant Cancer?. Cancer Cell.

[52] Denise C., Paoli P., Calvani M., Taddei M.L., Giannoni E., Kopetz S., Kazmi S.M.A., Pia M.M., Pettazzoni P., Sacco E. (2015). 5-Fluorouracil resistant colon cancer cells are addicted to OXPHOS to survive and enhance stem-like traits. Oncotarget.

